# Safety of Health Care Workers in a War Zone—A European Issue

**DOI:** 10.3389/fpubh.2022.886394

**Published:** 2022-03-30

**Authors:** Alpo Vuorio, Robert Bor

**Affiliations:** ^1^Department of Forensic Medicine, University of Helsinki, Helsinki, Finland; ^2^Mehiläinen Airport Health Centre, Vantaa, Finland; ^3^Centre for Aviation Psychology, London, United Kingdom; ^4^Royal Free Hospital, London, United Kingdom

**Keywords:** health care workers, war, mental health, safety, PTSD, COVID-19

## Introduction

According to the International Law and Rule 25, “*Medical personnel exclusively assigned to medical duties must be respected and protected in all circumstances. They lose their protection if they commit, outside their humanitarian function, acts harmful to the enemy*.” (1, 2). Despite this law, it is not uncommon that this aspect of international law is violated during conflicts. The International Committee of the Red Cross (ICRC) has emphasized that the problem of not protecting health care workers in a war zone is one of the most crucial yet overlooked humanitarian issues today (3), and conflict and war situations increase risk to harm of such workers (4, 5). Furthermore, the deleterious effects of war zone operations on health workers extends to material and supply shortages including breakdown in the supply of services (e.g., electricity, medicines, and equipment) which further increases stress on health care personnel (6). In the current conflict in Ukraine, the International Council of Nurses (ICN) has highlighted in a press release that the safety of health care workers during this conflict is paramount (7). This plea has been sent to both to both Ukrainian and Russian respective associations.

## Violence Against Healthcare Workers

In a recent systematic review, the authors examined 1,479 papers related to against health care violence in conflict zones (5). The articles covered different aspects of the problem such as evidence of the impact of attacks on health care personnel as well as violations of legal and human rights on such workers. The authors concluded that there is a need to broaden awareness of topic. The issues are diverse ranging from a risk of health care personnel being threatened, arrested or even jailed (5) to being forced to collaborate with one or the other side in a conflict by applying different levels of care to—or withholding it altogether from—wounded or sick people or combatants. Table 1 shows different violations against health care personnel. According to the above-mentioned review of the literature, most comes from Middle East and Asia, but among the literature is one study of attacks from Eastern Ukraine from the year 2014 (13). This article describes the damage caused to the public health infrastructure and analyses whether the damage caused was targeted or collateral.

**Table 1:** Violence against health care workers.

**Type of attack**	**References**
Beatings and shootings	(8)
Surveillance at work	(9)
Arrest, intimidation and threats	(10)
Obstruction of daily operations	(11)
Interference of obligations of impartial care	(12)

As a mitigation against violence toward to health professionals in conflict zones, the local community can be more directly engaged in ensuring secure access to health care (14), documenting and publicizing breaches rapidly (15). And also negotiating directly with conflict parties to ensure fair and safe provision of health services (16).

## Traumatic Stress Reaction and Health Care Workers

Risk for post-traumatic stress (PTSD) is well-documented among deployed military health care workers and is similar when compared to military personnel (17). It has been shown that trauma severity and additional life stress have an important impact in increasing the risk for PTSD (18). Social support increased the resilience and acted as a protective factor (19). Chronic PTSD has been reported in several studies among combat-exposed health care workers (20, 21). In a small study by Ravella (21) of nurses, almost 25% reported symptoms of PTSD several years after being caught up in an explosion. Based on their review, Gibbons et al. (17) call for more research into finding risk factors and protective factors for PTSD among health care personnel exposed to combat. Unfortunately, a supportive work environment does not eliminate the risk of PTSD among health care workers in conflict zones. It is possible that females may be more prone to risk for developing PTSD than males in these situations (22).

A very recent study regarding health care professionals' wellbeing under extreme circumstances comes from the conflict in Yemen (23). The authors carried out 43 facility-based health care worker interviews and additionally six group sessions. The complex security situation prevented health care personnel from carrying out their everyday work normally and increased their levels of stress. Specific themes related to coping were religious motivation, sense of duty and patriotism. There is clearly a lack of clear guidelines that address protective factors for mental health among healthcare workers under extreme stress, assuming that they are relevant or accessible (23, 24).

## Combined COVID-19 Pandemic and War Stress

Not surprisingly, there is little literature regarding the combined effect of the COVID-19 pandemic and war among health care workers. Elhadi et al. (25) studied the combined stress caused by the COVID-19 pandemic and the Libyan civil war among 532 health care workers. Of these health care personnel, 357 (67%) reported emotional exhaustion (EE Score ≥ 10), 252 (47%) reported depersonalization (DP score ≥ 10) and 121 (23%) reported a lower sense of personal accomplishment (PA score ≤ 10). The authors highlight the need to develop health care policies to protect them in unique threatening, hostile and stressful environments.

## Conclusion

The current conflict in Ukraine exposes many health care workers to severe stress. As refugees from the conflict arrive in different parts of the continent, almost all of Europe's health care systems will be challenged by the experience from conflict-exposed individuals. This will inevitably put a strain on health care staff some of whom may be traumatized vicariously. However, those who have encountered conflict and war directly, will likely suffer the greatest stress. The current situation requires the support of pan-European health professionals and investment in follow-up research and analysis of the situation. This is important, because The World Health Organization has confirmed “several” attacks on health care centers in Ukraine and is investigating others (26).

## Author Contributions

All authors listed have made a substantial, direct, and intellectual contribution to the work and approved it for publication.

## Conflict of Interest

The authors declare that the research was conducted in the absence of any commercial or financial relationships that could be construed as a potential conflict of interest.

## Publisher's Note

All claims expressed in this article are solely those of the authors and do not necessarily represent those of their affiliated organizations, or those of the publisher, the editors and the reviewers. Any product that may be evaluated in this article, or claim that may be made by its manufacturer, is not guaranteed or endorsed by the publisher.

## References

[1] ICRC. Geneva Conventions of 1949 and Additional Protocols, and their Commentaries. International Committee of the Red Cross (2021). Available online at: https://www.icrc.org/applic/ihl/ihl.nsf/vwTreaties1949.xsp (accessed February 26, 2022).

[2] International Humanitarian Law Database (IHLD),. (2021). Available online at: https://ihl-databases.icrc.org/customary-ihl/eng/docindex/v1_rul_rule25 (accessed February 26, 2022).

[3] ICRC. Health-Care Workers Suffer Attacks Every Single Week. (2018). Available online at: https://www.icrc.org/en/document/icrc-health-care-workers-suffer-attacks-every-single-week (accessed February 26, 2022).

[4] ICRC. Healthcare in Danger: Making the Case. (2011). Available online at: http://www.icrc.org/eng/resources/documents/publication/p4072.htm (accessed February 26, 2022).

[5] Haar RJ Read R Fast L Blanchet K Rinaldi S Taithe B. Violence against healthcare in conflict: a systematic review of the literature and agenda for future research. Conflict Health. (2021) 15:37. 10.1186/s13031-021-00372-7 33962623 PMC8103060

[6] PLoS Medicine Editors. Health care in danger: deliberate attacks on health care during armed conflict. PLoS Med. (2014) 11:e1001668. 10.1371/journal.pmed.1001668 24959856 PMC4068983

[7] International Council of Nurses. ICN Says Protection and Safety of Nurses and All Health Workers in Ukraine Is Paramount. (2022). Available online at: https://www.icn.ch/news/icn-says-protection-and-safety-nurses-and-all-health-workers-ukraine-paramount (accessed February 26, 2022).

[8] Crombé X Kuper J. War breaks out: interpreting violence on healthcare in the early stage of the south Sudanese civil war. J Humanit Aff. (2019) 1:4–12. 10.7227/JHA.012

[9] Michlig GJ Lafta R Al-Nuaimi M Burnham G. Providing healthcare under ISIS: a qualitative analysis of healthcare worker experiences in Mosul, Iraq between June 2014 and June 2017. Glob Public Health. (2019) 14:1414–27. 10.1080/17441692.2019.1609061 31034779

[10] Haar RJ Footer KH Singh S Sherman SG Branchini C Sclar J. Measurement of attacks and interferences with health care in conflict: validation of an incident reporting tool for attacks on and interferences with health care in eastern Burma. Conflict Health. (2014) 8:23. 10.1186/1752-1505-8-23 25400693 PMC4232629

[11] Eisenberg C Halperin D Hargreaves A Hubbard F Mittleberger J Palmisano J. Health and human rights in El Salvador. NEJM. (1983) 308:1028–9. 10.1056/NEJM198304283081713 6835310

[12] Bouchet-Saulnier F Whittall J. An environment conducive to mistakes? Lessons learnt from the attack on the Médecins Sans Frontières hospital in Kunduz, Afghanistan. Int Rev Red Cross. (2019) 100:1–36. 10.1017/S1816383118000619

[13] Bucley CJ Clem RS Herron ES. An assessment of attributing public health care infrastructure damage in the Donbas five years after Euromaiden: implications of Ukrainian state legitimacy. Eurasian Geogr Econ. (2019) 60:54–72. 10.1080/15387216.2019.1581634

[14] Footer KH Meyer S Sherman SG Rubenstein L. On the frontline of eastern Burma's chronic conflict–listening to the voices of local health workers. Soc Sci Med. (2014) 120:378–86. 10.1016/j.socscimed.2014.02.019 24666656

[15] Fast L Wille C. To stay or go? The complexities of providing healthcare in insecure environments. World Health Popul. (2016) 16:38–42. 10.12927/whp.2016.24674 27358018

[16] Terry F. Violence against health care: insights from Afghanistan, Somalia, and the Democratic Republic of the Congo. Int Rev Red Cross. (2013) 95:23–39. 10.1017/S1816383113000581

[17] Gibbons SW Hickling EJ Watts DD. Combat stressors and post-traumatic stress in deployed military healthcare professionals: an integrative review. J Adv Nurs. (2012) 68:3–21. 10.1111/j.1365-2648.2011.05708.x 21635285

[18] Brewin CR Andrews B Valentine JD. Meta-analysis of risk factors for posttraumatic stress disorder in trauma-exposed adults. J Consult Clin Psychol. (2000) 68:748–66. 10.1037/0022-006X.68.5.748 11068961

[19] Sweat M Snow D Eisenbrandt LG. Vietnam nursing: the experience lives on. Interview by Margaret Ann La Salle. Mil Med. (2000) 165:641–6. 10.1093/milmed/165.9.641 11011531

[20] Norman EM. Post-traumatic stress disorder in military nurses who served in Vietnam during the war years 1965-1973. Mil Med. (1988) 153:238–42. 10.1093/milmed/153.5.238 3138564

[21] Ravella PC. A survey of U.S. Air Force flight nurses' adaptation to service in Vietnam. Aviat Space Environ Med. (1995) 66:80–83. 7695558

[22] Bangasser DA Eck SR Ordoñes Sanchez E. Sex differences in stress reactivity in arousal and attention systems. Neuropsychopharmacology. (2019) 44:129–39. 10.1038/s41386-018-0137-2 30022063 PMC6235989

[23] Elnakib S Elaraby S Othman F BaSaleem H Abdulghani AlShawafi NA Saleh Al-Gawfi IA. Providing care under extreme adversity: the impact of the Yemen conflict on the personal and professional lives of health workers. Soc Sci Med. (2021) 272:113751. 10.1016/j.socscimed.2021.113751 33588206 PMC7938221

[24] van Ommeren M Hanna F Weissbecker I Ventevogel P. Mental health and psychosocial support in humanitarian emergencies. East Mediterr Health J. (2015) 21:498–502. 10.26719/2015.21.7.498 26442890

[25] Elhadi M Msherghi A Elgzairi M Alhashimi A Bouhuwaish A Biala M. Burnout syndrome among hospital healthcare workers during the COVID-19 pandemic and civil war: a cross-sectional study. Front Psychiatry. (2020) 11:579563. 10.3389/fpsyt.2020.579563 33362600 PMC7759513

[26] Reuters. Ukraine Health Centres Have Been Attacked, WHO Chief Says. (2022). Available online at: https://www.reuters.com/article/ukraine-crisis-russia-who-idUSL5N2V9047 (accessed February 26, 2022).

